# Neuro-dermatological association between psoriasis and depression: an immune-mediated inflammatory process validating skin-brain axis theory

**DOI:** 10.3934/Neuroscience.2021018

**Published:** 2021-03-10

**Authors:** Shahzaib Maqbool, Arham Ihtesham, Muhammad Nadeem Langove, Sara Jamal, Tabdar Jamal, Hafiz Abu Safian

**Affiliations:** 1 Department of Medicine, Rawalpindi Medical University, Pakistan; 2 Department of Haematology, Resident Haematology atomic energy Islamabad, Pakistan; 3 Department of Radiology, Resident Radiology Kahuta Research Laboratory (KRL) hospital, Pakistan

**Keywords:** depression, neuro-dermatology, psoriasis, skin-brain axis

## Abstract

**Objective:**

Our study's motive was to recognize various immune-mediated inflammatory processes involved in the pathogenesis of depression and psoriasis and interlink between them based on inflammatory mediators.

**Methods:**

A careful and comprehensive literature search was done through various databases like PubMed, Google Scholar, and EBSCO. A total of 56 studies were included in our study after careful screening.

**Results:**

The immune-mediated inflammatory process was significantly associated with the pathogenesis of both depression and psoriasis. Most of the inflammatory markers involved in Psoriasis (TNF-α, IL-2, IL-6, IL-23, IL-1β, IL-10), and increased serotonin transporters (5-HTT) were also found in the pathogenesis of depression, showing the immune-inflammatory linkage between psoriasis and major depression. Based on immune chemistry, the levels of CD2^+^, CD4^+^, CD8^+^ T-lymphocytes were also found to be raised in both depression and psoriasis, validating their relationship. Hyperactivity of HPA-axis was also found another interlink between them along with reduced melatonin amount.

**Conclusions:**

According to various studies, the neuro-dermatological association between psoriasis and depression is significant. Different immune markers involved in the pathogenesis of depression and psoriasis also show the bidirectional association between them. However, this association between psoriasis and depression is positively correlated, but more work is required to answer why all depressed patients fail to develop psoriasis and why all psoriatic patients fail to develop depression.

## 1. Introduction

A subdivision of medicine that elaborates the outcome of social, psychological, and behavioural factors on the bodily process and how they are interconnected and how they affect the quality of life is called psychosomatic medicine [1]. This article aimed to assess psoriasis and depression's association under the lights of published literature concerning unique terminology known as psycho-dermatology [2]. Psycho-dermatology is also a sub-branch of medicine that explains the association of the human nervous system and skin and its appendages [3]. The purpose of highlighting the association between psoriasis and depression is that only limited studies have been done on this specific subject to justify this association. Though studies show a significant association between psoriasis and depression, this subject still requires more insight to strengthen this concept of bidirectionality.

Psoriasis is described as a chronic inflammatory disease leading to multiple erythematous and plaques of silvery-White colour involving most commonly extensor surfaces like elbows and knees. However, it can involve whole body surfaces, as well [4]. Psoriasis's prevalence is on the rise, affecting 1–3% of the world population, which accounted for almost 125 million people worldwide. However, its prevalence varies from region-wise distribution affecting 0.91% of the population in the United States and on the extreme side in Norway, affecting 8.5% of the population manifesting the consortium between Psoriasis and geographical area [5]. Psoriasis is regarded as an immune-mediated inflammatory disorder associated with various interleukin and cytokine release [6]. Psoriasis is generally considered a genetically induced multifactorial disease associated significantly with environmental triggers, family history, physical, psychological factors, sometimes corticosteroid withdrawal, and infectious causes such as streptococcal infections that can lead to guttate psoriasis [7],[8]. Psoriasis is generally known as a dermatological problem that can lead to depression; however, this concept of unidirectional association between psoriasis and depression is getting obsolete over the past few years. The studies show a unique association named bidirectional association, showing that if psoriasis leads to depression, it is also the depression that can lead to psoriasis through an immune-mediated inflammatory process known as psycho-dermatology [9]. This thorough review highlights the psycho-dermatological linkage between major depression and psoriasis, also known as Psoriasis Vulgaris.

## 2. Materials and methods

**Figure 1.**
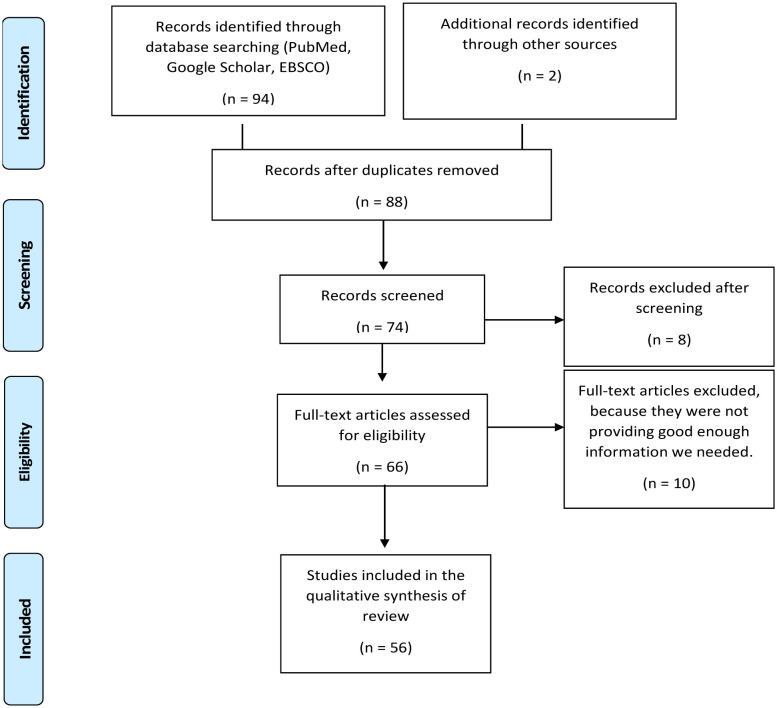
Prisma flow chart showing the selection of studies.

This is a systematic review for exploring the immune-mediated inflammatory association between psoriasis and significant depression. The literature was done using PubMed, Google Scholar, and EBSCO search engines using psoriasis's general keywords and significant depression. A total of 1470 studies were found using search terms of depression and psoriasis, but after searching the specific keywords related to our study aims and objectives, we found 94 studies of our interest with two studies from other sources. Some of these studies were duplications and were excluded from our study. A total of 56 studies were finally included. Throughout this process of selection of studies, PRISMA guidelines were followed and presented in the form of a PRISMA flow chart (Figure 1).

## 3. Psoriasis and depression: a skin-brain axis theory

The most common psychological factors associated with psoriasis are stress, anxiety, and depression, also known as major depression [10]. However, to justify this psycho-cutaneous association, various theories have been put forward, such as demoralized attitude towards treatment failure, decreased quality of life, and restricted social interaction, which ultimately worsens the disease symptoms and reduced adherence towards standard health care [11],[12]. Despite incredible work explaining this psycho-cutaneous association's true nature, some studies are paving a new way towards a mechanistic approach for validating this association between psoriasis and depression through an immune-mediated inflammatory process, also known as immune-mediated psycho-dermatology [13]. Various studies show a statistically significant association between various psychological parameters like stress and depression with psoriasis but the cause and the effect that is still not clear [11],[14]. Besides, studies also show that efficacious treatment of stress and depression can lead to a decrease in Psoriasis severity [15]. However, on the flip side, effective psoriasis treatment is also showing marked improvement in the above mentioned psychological symptoms [16]–[18]. However, now the question is which mechanism is involved in this pathophysiology and how it happens so? This study aimed to explore various mechanisms involving the conditions mentioned above.

### 3.1. The immune-mediated inflammatory process involved in psoriasis: an immune-dermatological component of skin-brain axis theory

Psoriasis is generally regarded as a multifactorial disease associated with various genetic and environmental factors that provoked immune-mediated inflammatory processes in various organs via Th 1 and Th 17 induced pathways [19],[20]. The most commonly affected body organs are joints (knee, hip, elbow, and hands) with an arthritic presentation called psoriatic arthritis, along with skin and CNS involvement leading to depression and psoriatic spondylitis [20]. With the advances in scientific methods and measures, various studies are also depicting dendritic cells' role in the initiation of inflammatory response mediated principally by IL-23/IL-17 cytokine axis and through the production of TNF-α and activation of myeloid dendritic cells [21]. The most crucial role played by these dendritic cells, as mentioned above, is to cause infiltration of various inflammatory infiltrates (T lymphocytes, neutrophil granulocytes, monocytes, macrophages) into the upper dermis epidermis and throughout the dermo-epidermal junction. Inflammatory myeloid dendritic cells release IL-23 and IL-12 to activate Th17, Th1, and Th22 cells, causing the production of psoriatic cytokines IL-17, IL-22, INF-γ, and TNF leading to psoriasis [22],[23]. The activated Th1 cells are associated with increased production of INF-γ that, on the other side, leads to the abundant production of chemokines (CXCL9, CXCL10, and CXCL11) from keratinocytes, that further recruit more Th1 cells. Similarly, activation of Th17 is found to be associated with increased production of IL-17, which further activates abundant production of CCL20, CXCL1, CXCL2, and CXCL8/IL-8 leading to recruitment of neutrophils into the skin [24].

Dendritic cells are considered antigen-presenting cells that have a paramount role in regulating the body's immune system through innate and adaptive immunity. Various types of dendritic cells are associated with psoriasis' pathogenesis, including Langerhans cells, plasmacytoid dendritic cells, and dermal myeloid DC [25]. The role of plasmacytoid and dermal myeloid is considered an integral component in the immunopathogenesis of psoriasis. Activated plasmacytoid DC is associated with increased production of INF-α, leading to initiation of an immune cascade, followed by conversion of plasmacytoid DC into dermal myeloid DC, leading to stimulation of Th1 and Th17 cells [26],[27]. Similarly, the commonly involved interleukins leading to psoriasis are IL-6, IL-13, IL-17, IL-23, IL-22, and IL-1β. According to various published literature, another set of cytokines is also involved in this immune-mediated psoriasis, known as the type-1 cytokine profile involving IL-2, INF-γ, and finally TNF-α and intercellular adhesion molecule (ICAM). All these chemical mediators are considered the mainstream of the immune-mediated inflammatory process of psoriasis because they exert a direct effect on keratinocytes and cause the release of abundant chemokines leading to an exaggerated inflammatory response, and ultimately psoriasis through hyperproliferation of keratinocytes and abnormal retention of nuclei in stratum corneum [28],[29]. Another key mediator that is also now considered to be an interlink between psoriasis and various psychological disorders is called a brain-derived neurotrophic factor (BDNF); however, the level of (BDNF) in the blood is found to be reduced in both psoriasis and depression, validating the interlink between disorders as mentioned earlier [30].

### 3.2. The immune-mediated inflammatory process involved in depression: neuro-inflammatory component of the skin-brain axis

Major depression is considered a leading cause of disease burden worldwide, affecting almost 4.4% of the world population [31]. The association between depression and the immune-mediated inflammatory process is well known in the scientific community and well-validated by various published works. Various pro-inflammatory cytokines and interleukins are associated with major depression, including increased prostaglandin E2 and C-reactive proteins (CRP) levels, similar levels of various interleukins IL-2, IL-6, and IL-1β are also found to elevate along with TNF-α [32],[33]. The increased levels of the aforementioned inflammatory markers are significantly associated with an increased risk of disease severity and morbidity. Some studies also show that inflammatory cytokines, particularly TNF-α, are significantly associated with serotonin transporters activity (5-HTT), showing that increased concentration of TNF-α in the body also causes increased 5-HTT activity in the body and vice versa [9]. This concept is firmly validated by using TNF-α inhibitors like infliximab and etanercept, which caused a significant reduction in serotonin transporters activity levels and increased serotonin availability levels in the body. However, the immune mechanism is not limited to these mediators, as mentioned above, because several immune dysregulation agents are also associated with significant depression-like CXCL 10/IP-10. As evident from a published work, its levels are increased in major depression, and antidepressants are associated with reducing CXCL.10/IP-10 levels in the body, validating this mediator's role in the development of major depression [34].

Patients with major depression have been repeatedly observed to have increased inflammatory cytokines, chemokines, and acute-phase proteins such as C-reactive proteins (CRP) in peripheral blood and cerebrospinal fluid (CSF) [35]. The inflammatory process is considered a cardinal feature of major depression; however, studies show the involvement of the inflammatory process even in mildly depressed patients [36]. Studies are showing a significant association between depressive symptoms like fatigue, impaired sleep, cognitive impairment, and various inflammatory markers [37]. On peripheral blood analysis, IL-1, IL-6, and TNF-α are the most common cytokines involved in the pathogenesis of major depression [38]. Studies also demonstrate that administrations of various acute and chronic cytokines or cytokines inducers like lipopolysaccharide (LPS) and vaccination were significantly associated with behavioural changes causing depression and anxiety [39]. Studies are also indicating that peripheral administration of INF-α for treatment of hepatitis C led to increased levels of INF-α in cerebrospinal fluid (CSF) and increased levels of IL-6, and chemokine such as monocyte chemoattractant protein 1 (MCP-1) [40]. Some studies were also demonstrating the impact of LPS, administered peripherally, associated with cognitive impairment and increased amount of TNF-α, IL-1, and the reduced hippocampal expression of brain-derived neurotrophic factors (BDNF) associated receptors leading to reduced hippocampal neurogenesis [41].

### 3.3. The immune-mediated inflammatory process that interlink psoriasis and depression: a bidirectional association validating skin-brain axis theory

After carefully describing the immune-inflammatory processes involved in the pathophysiology of depression and psoriasis, we concluded that major depression and psoriasis are significantly associated with each other validating their directionality evident from the presence of similar cytokines interleukins and other mediators involving pathologies as mentioned above. However, to strengthen this concept of bidirectionality, we believe that still more studies are required. Many other studies are still proving this bidirectional association, as evident from the published literature that the pro-inflammatory cytokines involved in Psoriasis' pathogenesis are likely to cause hyperactivation of the Hypothalamic-Pituitary-Adrenal axis leading to major depression. This hyperactivity of the Hypothalamic-Pituitary-Adrenal axis also leads to decreased serotonin levels, which is another causative factor in developing major depression [42],[43]. After successfully displaying the immune-inflammatory process, another interlink between significant depression and psoriasis has also been identified by many scholars called melatonin. As evident from the literature, melatonin levels are decreased in both depression and psoriasis, favouring the bidirectional association [44],[45]. The proposed mechanism involved in psoriasis and depression's development is the activation of the melatonin-mediated immune-inflammatory process leading to elevated TNF-α, IL-6, and IL-8, respectively [46],[47].

The studies are also suggesting that major depression and psoriasis have a high impact on both genetic and immunological parameters, as evident from a study showing that levels of CD2^+^, CD4^+^, CD8^+^ cells were found to be high in both psoriasis and depression with a markedly increased ratio of CD4^+^/CD8^+^ cells [48]. However, an interesting study involving genetic association between psoriasis and depression was conducted by Demirhan et al., was showing the numerical alteration at chromosomes 8, 15, 21, 22, Y, and X, with chromosome 8 being the most commonly affected. The structural changes were comprised of duplication, translocation, deletion, and breaks, with a focus on loci on del (1) (q12–q23), t (2; 22) (p14; 13), and dup (10) (q26), with the presence of susceptible genes of psoriasis and major depression on these loci [49]. So, all the above-mentioned evidence-based studies depict psoriasis' directionality and significant depression, but the quest for knowledge is not ended yet; there are still more shreds of evidence that need to be explored to justify this association with a more precise description.

**Table 1.** Types of studies involved in this systematic review.

Type of studies	No of studies
Cross-sectional	4
Longitudinal	1
Review articles	6
Case-control	1
Clinical trial	1

**Table 2.** Details of studies included in our systematic review.

Author/year of publication	Country	Study types	Population included	Sample size	Diagnostic criteria	Comments
Jose et al. [50], 2019	Spain	Case-control	Seventy subjects who were suffering from moderate to severe psoriasis and 140 healthy controls were included.	210	Un-specified	Psoriasis is considered a chronic inflammatory process manifested as Erythematous, silvery-white plaques. It is significantly associated with psychological symptoms of depression, anxiety, and stress with the reduced overall quality of life.
Huerta et al. [8], 2007	UK	Cross-sectional	Those who were newly diagnosed with psoriasis and were free of any malignancy	3994 psoriatic patients out of 10,000 random population	Not specified	The estimated incidence rate of psoriasis was 14 percent per 10,000 population as determined in this study. All those who were already suffering from some dermatological disease within the last year were at more risk of developing Psoriasis. Similarly, smoking was found to be an independent risk factor for psoriasis.
Nicholas et al. [51], 2017	Canada	Review	Not applicable	Not applicable	Not applicable	Psoriasis is a chronic inflammatory condition associated significantly with depression and suicidal ideation. The most important risk factors that are associated with depression are age and severity of psoriasis. The treatment of psoriasis is associated with decreased intensity of depressive symptoms. The young people were showing more preponderance towards depression after being suffering from psoriasis.

Fortune et al. [16], 2002	USA	Longitudinal Study	All the patients who have psoriasis and attending outpatient department for simple psoriasis treatment (n = 53), and those willing to undergo the psoriasis management Program (n = 40).	93	DMS-IV	According to this study, two comparison groups were formulated. Firstly, those who were taking treatment of psoriasis alone and those involved in the psoriasis management program. The study results showed a significant reduction in psoriasis severity, level of anxiety, depression, and stress among those who were taking programmed psoriasis management compared to those taking alone psoriasis treatment.
Fortune et al. [16], 2002	USA	Longitudinal Study	All the patients who have psoriasis and attending outpatient department for simple psoriasis treatment (n = 53), and those willing to undergo the psoriasis management Program (n = 40).	93	DMS-IV	According to this study, two comparison groups were formulated. Firstly, those who were taking treatment of psoriasis alone and those involved in the psoriasis management program. The study results showed a significant reduction in psoriasis severity, level of anxiety, depression, and stress among those who were taking programmed psoriasis management compared to those taking alone psoriasis treatment.
Uyemura et al. [29], 1993	USA	Cross-sectional study	All those who were diagnosed with a case of psoriasis based on skin biopsy were included.	Not specified	DSM-IV	The immune-inflammatory profile of psoriasis was identified in this study. According to study findings, the type-1 profile of cytokines (TNF-α, IL-2, and INF-γ) was recognized as the leading inflammatory and INF-γ) was recognized as the leading inflammatory markers of psoriasis. The over-expression of VCAM-1, ICAM-1, was also observed on skin biopsies of diseased persons.
Keller et al. [42], 2017	UK	Clinical trial	Fifty-nine patients suffering from psychotic major depression and 58 patients who were facing non-psychotic depression were evaluated for their HPA-axis activity.	117	DSM-IV	This study aimed to assess the Hypothalamic-Pituitary-Adrenal axis's involvement in various psychological disorders such as depression and cognitive defects based on cortisol levels in the blood. The study findings showed that the hyperactivity of the HPA-axis among those suffering from significant depression, increased cortisol levels, and cognitive impairment was negatively correlated with the HPA-axis and increased cortisol levels in the blood.
Aleem et al. [52], 2018	USA	Review	Not applicable	Not applicable	Not applicable	Psoriasis and depression were found to be significantly associated with each other. This association's bi-directionality was justified through the similarity of inflammatory markers involved in the pathogenesis of depression and psoriasis. The most common inflammatory markers that are elevated in this association were TNF-α, IL-1, IL-22, IL-17, IL-1β and IL-6, IL-8, raised CRP, and some genetic factors also found in this association.
Kannan et al. [53], 2013	USA	Review	Not applicable	Not applicable	Not applicable	This study was used for assessing the usefulness of anti-TNF-α (infliximab, adalimumab) in the treatment of depression and psoriasis. However, they were found to be effective in treating depression and associated with chronic inflammatory conditions.
Komiya et al. [54], 2020	Japan	Review	Not applicable	Not applicable	Not applicable	This comprehensive review was depicting the role of inflammatory mediators and HPA-axis involvement in the pathogenesis of psoriasis. The increased IL-23, IL13, IL-10 were aggravating factors in the itching process of psoriasis. Simultaneously, over-activity of the HPA-axis was also observed that was increasing CRH and α-MSH levels, which were implicated in the induction of itching process through various receptor-like CRHR1, MC1R, MC5R.
Moon et al. [55], 2013	UK	Review	Not applicable	Not applicable	Not applicable	Stress is regarded as an immune-mediated trigger for activating various neurohormones through the HPA-axis via upregulation of CRH, adrenocorticotropic hormones, and glucocorticoids. Glucocorticoids cause inhibition of IL-12, INF-γ, TNF-α through antigen-presenting cells and Th1 cells, and up-regulation of IL-4, IL-10, and IL-13 through Th2 cells. However, cortisol secretion exerts an immunosuppressive effect by shifting the immune response from Th1 to Th2 cells. This inflammatory response is associated with increased activity of mast cells histamine that causes increased levels of IL-6, IL-10 as occur in psoriasis, and causes an increased level of CRH that leads to induction of sickness behaviour and depressive symptoms.
Motivala et al. [56], 2005	USA	Cross-sectional study	Twenty-two acutely depressed patients and 18 well-matched controls were selected	40	DSM-IV	After careful matching in terms of gender and age distribution with the control group, it was found that the levels of IL-6 and ICAM-1 were significantly high during night time. The REM density and sleep latency were found to be associated with both ICAM-1 and IL-6 levels. However, both (sleep latency and REM density) are recognized as better predictors of IL-6 and ICAM-1.
Kartha et al. [46], 2014	India	Cross-sectional Study	Those diagnosed with psoriasis (n = 36), and their matched healthy controls (n = 36)	72	DSM-IV	The melatonin levels measured at night in serum showed a significant decline in psoriasis cases compared with age and gender-matched controls; melatonin is an anti-inflammatory molecule. Its low levels are significantly associated with pruritic episodes, which may cause koebnerization that further intensifies psoriasis severity. The properties of melatonin can be modulated to reduce the release of different pro-inflammatory cytokines and reduce the depression severity with different antidepressants. This concept may open the gates for a new generation of antidepressants in the future for effective management of depression with a significant reduction of morbidity in patients with psoriasis.
Esposito et al. [47], 2010	Italy	Review	Not applicable	Not applicable	Not applicable	Many studies are showing the anti-inflammatory effects of melatonin through its immunomodulatory property. It causes a reduction in inflammatory cytokines and ICAM, VCAM levels in the blood. Many studies are also manifesting its role in neurodegenerative disorders as well through its antioxidative activity. Its neuroprotective role is significant in preventing oxidative brain damage from free radical injuries through stimulation of various antioxidant enzymes.

Note: HPA-axis: Hypothalamic-Pituitary-Adrenal axis; VCAM-1: vascular cell adhesion molecule-1; ICAM-1: intercellular adhesion molecule-1; DSM-IV: Diagnostic and Statistical Manual of Mental Disorders.

**Table 3.** Similarity in immune-inflammatory markers involved in the pathogenesis of psoriasis and major depression.

Immune-inflammatory mediators	Levels in Psoriasis	Levels in Depression
TNF-α, IL-1β, IL-6, IL-2, INF-Υ	Increased	Increased
Melatonin levels	Decreased	Decreased
CRP	Increased	Increased
BDNF	Decreased	Decreased
CXCL-10/IP-10	Increased	Increased
Serotonin Transporter Activity (5HTT)	Increased	Increased
CD2^+^, CD4^+^, CD8^+^ T-lymphocytes	Increased	Increased
CD4/CD8 ratio	Increased	Increased
Prostaglandins-E2	Increased	Increased

Note: CRP: C-reactive protein; BDNF: Brain derived neurotrophic factor.

## 4. Conclusions

Though very little work has been done to find the association between depression and psoriasis to prove it through skin-brain axis theory, some studies clearly show this association of bidirectionality. This association of psoriasis and depression is not just a coincidental finding; in actual, an immune-mediated inflammatory process is involved in proving this bidirectional association. The most commonly involved inflammatory markers observed in the pathogenesis of both depression and Psoriasis are IL-6, IL-17, IL-13, IL-23, IL-10, IL-1β, and type A cytokines (TNF-α, IL-2, INF-γ) through Th1 and Th17 lymphocytes mediated processes. This pro-inflammatory cytokine production was also associated with HPA-axis over-activation with serotonin transporters' upregulation (5-HTT) and reduced serotonergic neurotransmitters in the body's depression. However, melatonin level was also found to be on the lower side in both depression and psoriasis, validating the bidirectional association. However, why every depressed patient never develops psoriasis, and why every patient with psoriasis is not always depressed, this mastery still needs more discussion and debate.

## References

[1] Strain JJ (2017). Globalization of psychosomatic medicine. Gen Hosp Psychiatry.

[2] Brown GE, Malakouti M, Sorenson E (2015). Psychodermatology. Adv Psychosom Med.

[3] Leon A, Levin EC, Koo JY (2013). Psychodermatology: an overview. Semi Cutan Med Surg.

[4] Karam RA, Zidan HE, Khater MH (2014). Polymorphisms in the TNF-α and IL-10 gene promoters and risk of psoriasis and correlation with disease severity. Cytokine.

[5] Parisi R, Symmons DP, Griffiths CE (2013). Global epidemiology of psoriasis: a systematic review of incidence and prevalence. J Invest Dermatol.

[6] Ovčina-Kurtović N (2016). Significance of cytokine as a predictor for psoriasis.

[7] Chong HT, Kopecki Z, Cowin AJ (2013). Lifting the silver flakes: the pathogenesis and management of chronic plaque psoriasis. Biomed Res Int.

[8] Huerta C, Rivero E, Garcia Rodriguez LA (2007). Incidence and risk factors for psoriasis in the general population. Arch Dermatol.

[9] Krishnadas R, Nicol A, Sassarini J (2016). Circulating tumour necrosis factor is highly correlated with brainstem serotonin transporter availability in humans. Brain Behav Immune.

[10] Hardy GE, Cotterill JA (1982). A study of depression and obsessionality in dysmorphophobic and psoriatic patients. Br J Psychiatry.

[11] Gupta MA, Gupta AK, Schork NJ (1994). Depression modulates pruritus perception: a study of pruritus in psoriasis, atopic dermatitis, and chronic idiopathic urticaria. Psychosom Med.

[12] Renzi C, Picardi A, Abeni D (2002). Association of dissatisfaction with care and psychiatric morbidity with poor treatment compliance. Arch Dermatol.

[13] Buske-Kirschbaum A, Ebrecht M, Kern S (2006). Endocrine stress responses in TH1-mediated chronic inflammatory skin disease (psoriasis vulgaris)—do they parallel stress-induced endocrine changes in TH2-mediated inflammatory dermatoses (atopic dermatitis). Psychoneuroendocrinology.

[14] Gupta MA, Gupta AK, Kirkby S (1989). A psychocutaneous profile of psoriasis patients who are stress reactors. A study of 127 patients. Gen Hosp Psychiatry.

[15] Matiushenko VP, Kutasevych YF, Havryliuk OA (2020). Effectiveness of psychopharmacotherapy in psoriasis patients with associated anxiety and depression. Dermatol Ther.

[16] Fortune DG, Richards HL, Kirby B (2002). A cognitive-behavioural symptom management programme as an adjunct in psoriasis therapy. Br J Dermatol.

[17] Redighieri IP, Maia T, Nadal MA (2011). Erythrodermic psoriasis with regression after prophylaxis with isoniazid and antidepressant therapy: case report. An Bras Dermatol.

[18] Menter A, Augustin M, Signorovitch J (2010). The effect of adalimumab on reducing depression in patients with moderate to severe psoriasis: a randomized clinical trial. J Am Acad Dermatol.

[19] Griffiths CE, Barker JN (2007). Pathogenesis and clinical features of psoriasis. Lancet.

[20] Gelfand JM, Gladman DD, Mease PJ (2005). Epidemiology of psoriatic arthritis in the population of the United States. J Am Acad Dermatol.

[21] Palfreeman AC, McNamee KE, McCann FE (2013). New developments in the management of psoriasis and psoriatic arthritis: a focus on apremilast. Drug Des Devel Ther.

[22] Lowes MA, Suárez-Fariñas M, Krueger JG (2014). Immunology of psoriasis. Annu Rev Immunol.

[23] Van der Fits L, Mourits S, Voerman JS (2009). Imiquimod-induced psoriasis-like skin inflammation in mice is mediated via the IL-23/IL-17 axis. J Immunol.

[24] Nograles KE, Zaba LC, Guttman-Yassky E (2008). Th17 cytokines interleukin (IL)-17 and IL-22 modulate distinct inflammatory and keratinocyte-response pathways. Br J Dermatol.

[25] Zaba LC, Krueger JG, Lowes MA (2009). Resident and “inflammatory” dendritic cells in human skin. J Invest Dermatol.

[26] Chu CC, Di Meglio P, Nestle FO (2011). Harnessing dendritic cells in inflammatory skin diseases. Semin Immuno.

[27] Nestle FO, Conrad C, Tun-Kyi A (2005). Plasmacytoid predendritic cells initiate psoriasis through interferon-alpha production. J Exp Med.

[28] Nestle FO, Kaplan DH, Barker J (2009). Psoriasis. N Engl J Med.

[29] Uyemura K, Yamamura M, Fivenson DF (1993). The cytokine network in lesional and lesion-free psoriatic skin is characterized by a Thelper type 1 cell-mediated response. J Invest Dermatol.

[30] Brunoni AR, Lotufo PA, Sabbag C (2015). Decreased brain-derived neurotrophic factor plasma levels in psoriasis patients. Braz J Med Biol Res.

[31] Mora C, Zonca V, Riva MA (2018). Blood biomarkers and treatment response in major depression. Expert Rev Mol Diagn.

[32] Rosenblat JD, Cha DS, Mansur RB (2014). Inflamed moods: a review of the interactions between inflammation and mood disorders. Prog Neuropsychopharmacol Biol Psychiatry.

[33] McNally L, Bhagwagar Z, Hannestad J (2008). Inflammation, glutamate, and glia in depression: a literature review. CNS Spectr.

[34] Wong ML, Dong C, Maestre-Mesa J (2008). Polymorphisms in inflammation-related genes are associated with susceptibility to major depression and antidepressant response. Mol Psychiatry.

[35] Miller AH, Maletic V, Raison CL (2009). Inflammation and its discontents: the role of cytokines in the pathophysiology of major depression. Biol Psychiatry.

[36] Raison CL, Capuron L, Miller AH (2006). Cytokines sing the blues: inflammation and the pathogenesis of depression. Trends Immunol.

[37] Dantzer R (2004). Cytokine-induced sickness behaviour: a neuroimmune response to activation of innate immunity. Eur J Pharmacol.

[38] Mössner R, Mikova O, Koutsilieri E (2007). Consensus paper of the WFSBP Task Force on Biological Markers: biological markers in depression. World J Biol Psychiatry.

[39] Reichenberg A, Yirmiya R, Schuld A (2001). Cytokine-associated emotional and cognitive disturbances in humans. Arch Gen Psychiatry.

[40] Raison CL, Borisov AS, Majer M (2009). Activation of central nervous system inflammatory pathways by interferon-alpha: relationship to monoamines and depression. Biol Psychiatry.

[41] Wu CW, Chen YC, Yu L (2007). Treadmill exercise counteracts the suppressive effects of peripheral lipopolysaccharide on hippocampal neurogenesis and learning and memory. J Neurochem.

[42] Keller J, Gomez R, Williams G (2017). HPA axis in major depression: cortisol, clinical symptomatology and genetic variation predict cognition. Mol Psychiatry.

[43] Brunoni AR, Santos IS, Sabbag C (2014). Psoriasis severity and hypothalamic-pituitary-adrenal axis function: results from the CALIPSO study. Braz J Med Biol Res.

[44] Mozzanica N, Tadini G, Radaelli A (1988). Plasma melatonin levels in psoriasis. Acta Derm Venereol.

[45] Quera Salva MA, Hartley S, Barbot F (2011). Circadian rhythms, melatonin and depression. Curr Pharm Des.

[46] Kartha LB, Chandrashekar L, Rajappa M (2014). Serum melatonin levels in psoriasis and associated depressive symptoms. Clin Chem Lab Med.

[47] Esposito E, Cuzzocrea S (2010). Anti-inflammatory activity of melatonin in central nervous system. Curr Neuropharmacol.

[48] Tohid H, Aleem D, Jackson C (2016). Major Depression and Psoriasis: A Psychodermatological Phenomenon. Skin Pharmacol Physiol.

[49] Demirhan O, Demirbek B, Tunç E (2012). Identification of chromosome abnormalities in screening of a family with manic depression and psoriasis: predisposition to aneuploidy. Asian J Psychiatr.

[50] Martínez-Ortega JM, Nogueras P, Muñoz-Negro JE (2019). Quality of life, anxiety and depressive symptoms in patients with psoriasis: A case-control study. J Psychosom Res.

[51] Nicholas MN, Gooderham M (2017). Psoriasis, Depression, and Suicidality. Skin Therapy Lett.

[52] Aleem D, Tohid H (2018). Pro-inflammatory Cytokines, Biomarkers, Genetics and the Immune System: A Mechanistic Approach of Depression and Psoriasis. Rev Colomb Psiquiatr.

[53] Kannan S, Heller MM, Lee ES (2013). The role of tumor necrosis factor-alpha and other cytokines in depression: what dermatologists should know. J Dermatolog Treat.

[54] Komiya E, Tominaga M, Kamata Y (2020). Molecular and Cellular Mechanisms of Itch in Psoriasis. Int J Mol Sci.

[55] Moon HS, Mizara A, McBride SR (2013). Psoriasis and psychodermatology. Dermatol Ther.

[56] Motivala SJ, Sarfatti A, Olmos L (2005). Inflammatory markers and sleep disturbance in major depression. Psychosom Med.

